# Sustainability Indicators Concerning Waste Management for Implementation of the Circular Economy Model on the University of Lome (Togo) Campus

**DOI:** 10.3390/ijerph16122234

**Published:** 2019-06-25

**Authors:** Lucía Salguero-Puerta, Juan Carlos Leyva-Díaz, Francisco Joaquín Cortés-García, Valentín Molina-Moreno

**Affiliations:** 1 Management Department-1, University of Granada, 18071 Granada, Spain; luciasalguero88@gmail.com; 2 Department of Chemical and Environmental Engineering, University of Oviedo, 33006 Oviedo, Spain; jcleyvadiaz@uniovi.es; 3 Faculty of Business and Management, Universidad Autónoma de Chile, 7500912 Santiago, Chile; franciscojoaquincortesgarcia@gmail.com

**Keywords:** circular economy, developing countries, energy, indicators, recycling, waste management

## Abstract

The circular economy aims to reduce the volume of waste generated in the world, transforming it into resources. The concept of indicator of circular economy was introduced to evaluate the improvement obtained regarding efficiency in terms of reduction, reuse and recycling of waste generated on the campus of the University of Lome (Togo). These indicators showed that 59.5% of the waste generated on the campus in 2018 could be introduced into the circular economy paradigm through composting, and 27.0% of the energy consumed could be replaced by clean energy obtained from biogas. The entire plastic fraction can be introduced into the circular economy paradigm by reusing plastic bottles and selling the rest in the port of the city. Thus, the income obtained could range from €15.5/day in 2018 to €34.5/day in 2027. Concerning old tires, 1.5% of the rubber needed to pave the entire roadway of the campus could be replaced by the waste generated by the tires currently existing there. Consequently, waste management on the campus could be controlled thanks to these indicators, and this could serve as a model for the rest of the country.

## 1. Introduction

Nowadays, humans exploit natural resources that produce waste. In Latin, “residuum” means “that which remains”, that is, a residue is what is discarded after taking advantage of something. Directive 2008/98/European Commission (EC) [1] on waste defines it as any substance or object that is discarded or intended to be discarded by its owner.

According to Zamarriego [2], the concept of “use and throw” appeared during the Industrial Revolution, when products began to follow the linear process of extraction–transformation–distribution–marketing–use–obsolescence. This is called a linear economy, which has proven to be unsustainable, causing a depletion of natural resources and variations in the composition and quantity of waste. In addition, it has led to an increase in the amount of waste in landfills, causing soil and water pollution, global warming, proliferation of pests, soil occupation, and destruction of the landscape [3]. Sustainable waste management tackles environmental and social issues that affect current and future generations. The sustainability analysis carried out by Stafford et al. [4] indicates that attention must be focused on environmental problems that are more relevant, from the development of theories such as functionality, ecology, and sustainable development. The circular economy constitutes a paradigm that will facilitate the transition of organizations to a more sustainable model.

This has forced the design of strategies aimed at reducing the volume of waste generated, paving the way for a circular economy model. According to the European Environment Agency [5], circular economy represents a fundamental alternative to the linear take-make-consume-dispose economic model that currently predominates. Circular economy proposes a scheme in which waste is replaced by subproducts. According to Directive 2008/98/EC [1] on waste, a subproduct is a substance or object resulting from a production process that can be used again in another production process without subsequent transformation, except the usual industrial practice, and without producing adverse impacts on human health. Thus, circular economy is characterized by the removal or mitigation of wastes and subproducts in different production processes, and if that elimination or reduction were not possible, this new paradigm considers that wastes and subproducts must be integrated into the same productive processes or others of similar or different nature with the aim of avoiding negative externalities and protecting the environment.

Among these change strategies, the principle of the “3Rs” appears: reduce, reuse, and recycle. The waste hierarchy lists different options for managing waste from an environmental perspective, from best (waste prevention) to worst (disposal). In this context, Directive 2008/98/EC [1] establishes two main objectives for EU waste legislation: waste prevention and development of a “recycling society.” Waste prevention has been and continues to be the first and most important objective of the EU waste management policy. Developing a recycling society implies reducing the environmental impact of resource use and improving the resource efficiency of such use. This last objective not only avoids producing waste, but also uses it as a resource. Braungart and McDonough [6] proposed a new way of interpreting environmentalism (the Next Industrial Revolution) in their book *Cradle to Cradle: Remaking the Way We Make Things*. Thus, circular economy interrelates with sustainability, seeking to keep the value of resources in the economy as long as possible, minimizing waste generation, transforming it into resources, and reintroducing these resources into production processes. For this, and according to Molina-Moreno et al. [7], it differentiates biological (biodegradable) nutrients from technological nutrients (designed for reuse or recycling).

**Figure 1:**
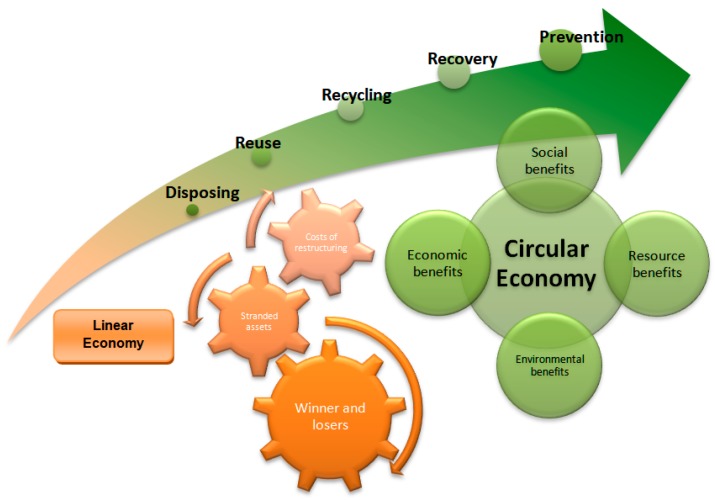
Transition from linear to circular economy model.

Waste management is currently carried out in the following order: production, presentation, collection, transport, and treatment. According to Argudo-García et al. [8], all waste should be reused, and for that, circular economy aims to minimize the elimination. Figure 1 shows the transition from a linear to a circular economy model.

To get a real circular economy model from the current linear economy model, the following steps must be taken: reuse, recycling, recovery, and prevention. Reuse is the first step to start the transition to the circular economy, but prevention is the most important step in this new paradigm.

Circular economy is an aspiration without borders. According to Reike et al. [9], in international politics, the urgency of closing materials loops is also more recently promoted by a consortium of global actors such as the Organization for Economic Co-operation and Development (OECD), the World Economic Forum (WEF), and the United Nations Environment Program (UNEP).

According to Quartey et al. [10], sustainable management of solid waste is a critical problem for developing countries with respect to climate change and greenhouse gas emissions, and also for the general welfare of the population. Developing countries lack sufficient infrastructure to process solid waste in an appropriate manner [11]. Preston and Lehne [12] show that in developing countries, the state of the environment and health outcomes have worsened because of a growing waste crisis.

According to Hassan et al. [13], the causes of the waste crisis could be a lack of waste segregation at the source, a lack of policies, failure of planning, inadequate training, a lack of awareness of the hazardous nature of every kind of waste, weak infrastructure, and a lack of suitable treatment technologies. Poor waste management is a key factor in the proliferation of diseases that may affect human health in urban settlements of developing countries [14].

As Matete and Trois [15] demonstrate, waste minimization can be introduced into emerging countries. According to Munguía et al. [16], changes in critical tasks in education, culture, and public policy are required in order to achieve “zero waste”. In light of this, one of the objectives of the new paradigm of the circular economy is to remove or mitigate wastes through a design that has as its main requirements the minimization of resources used in manufacturing and reduction of the energy balance of production, as well as decreased water and carbon footprints. On the other hand, if wisely managed, waste in developing countries represents a significant source of biomass, recycled materials, chemicals, energy, and revenue.

**Figure 2:**
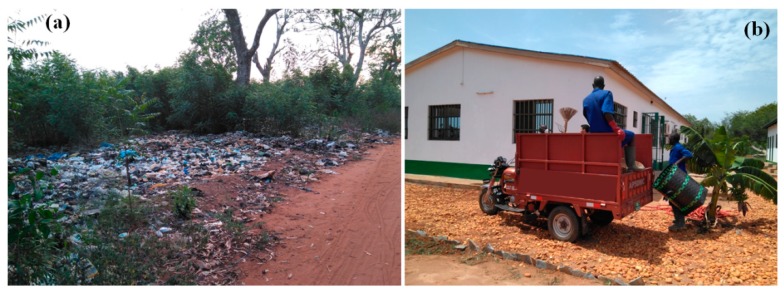
(**a**) Uncontrolled dump and (**b**) waste collection team on the campus of the University of Lome.

In Togo, and specifically on the campus of the University of Lome (the capital city), there is a great problem in the management of urban solid waste. There are uncontrolled dumps scattered throughout the country and, even more serious, on the university campus. Until recently, the waste generated in faculties and schools, administrative centers, and residence halls was thrown away throughout the campus without any control or management, to be burned afterward (Figure 2a).

According to Yukalang et al. [11], solutions to this problem could include developing appropriate policies and implementation plans, initiating a collection service that supports waste separation at the source, and educating the citizens of the campus and the government of the university. As Douti et al. [17] show, waste management institutions in developing countries face financial difficulties, understaffing, poor logistics (Figure 2b), road conditions, and social constraints.

Environmental contamination due to solid waste mismanagement is a global issue [18]. The main implemented waste treatments in developing countries are open dumping and open burning. Tan and Khoo [18] compared the potential environmental impacts of waste treatment options like landfilling, incineration, recycling and composting. In this regard, incineration imposes considerable harm to both environment and human health, especially for the burning of plastics, landfill gases and leachate, generating environmental damage (methane emissions, soil pollution, disease-transmitting insects…). However, composting hardly imposes any environmental damage, and recycling offers the best solution for environmental protection and improves human health. According to Ackerman [19] waste management can impact on climate change on five forms: landfill methane emissions, reduction in industrial energy use, energy recovery from waste, carbon sequestration in forest recycling paper, and energy used in long-distance transport of waste. In this regard, the Ministry of Environment and Natural Resources has signed an agreement with Climate Change Submit in 2015 to reduce greenhouse gas emissions, including the management of its waste among the measures.

Hoornweg and Bhada-Tata [20] categorize waste composition and origin as organic, including food scraps, yard waste (leaves, grass, brush) and wood; paper, including paper scraps, cardboard, newspapers, magazines, bags, boxes, wrapping paper, and telephone books; plastic, including bottles, packaging, containers, bags, lids, and cups; glass, including bottles, broken glassware, light bulbs, and colored glass; metals, including cans, foil, non-hazardous aerosol cans, and appliances (white goods); and others, including old tires, textiles, leather, rubber, e-waste, appliances, ash, and other inert materials. On the campus of the University of Lome, organic materials, paper, plastic, and old tires are the most common waste.

Given the large population studying and working on the campus (more than 50,000 people), the university community needs to manage urban solid waste in a more efficient manner. Despite the fact that it is not the main activity of the university, this initiative would allow it to design a specific strategy of waste management in order to comply with three objectives within the social responsibility programs of the University of Lome: improving health and environmental conditions on the campus, proposing research projects related to waste recycling and environmental sustainability, and promoting environmental awareness in the university community. As Molina-Moreno et al. [21] show, introducing the circular economy model would lead to the use of waste as a resource, and also to a cleaner environment.

Ayodele et al. [22] show how it is possible to recycle, reuse, and obtain energy from the fractions of waste classified by Hoornweg and Bhada-Tata [20]. In this way, the campus inhabitants would achieve sustainable management and the University of Lome would be able to generate income and save energy. In addition, integrating the concept of circular economy on the campus could be considered an effective strategy that, not only reduces post-consumer waste pollution, but also mitigates greenhouse gas (GHG) emissions [23]. This is very important, as the University of Lome (the main university of Togo) serves as a model for the rest of the country and, consequently, would be providing value at a national level so that Togo could pave the way for a circular economy model in the future.

According to Kavuza et al. [24], a reliable management system is necessary for high-quality and sustainable management of solid waste. The benefits of adopting a waste management plan on the campus are environmental benefits, reduced landfilling, reduced waste on site, and prevention of soil pollution.

The global concern for conserving the natural environment and the search for sustainable development have led to the development of systems of environmental indicators [25]. According to Molina-Moreno et al. [26], it is necessary to design indicators that allow assessment of the improvement obtained regarding efficiency in terms of reduction, reuse, and recycling of waste generated in the linear economy model. It is possible to determine if a process is closed to the circular economy model by means of the circular economy indicators [27].

The main objective of this work is to design indicators of circular economy for waste management on the campus of the University of Lome by using data collected in 2017 concerning the quantification and characterization of urban solid waste generated on the campus. This may facilitate measuring the progress of waste management on campus, focusing on the fact that energy can be extracted from any fraction of waste and that waste can be reused or recycled. Specifically, indicators of circular economy for organic, paper, textile, plastic and old tire fractions were obtained for 2018. Moreover, this paper shows a temporal evolution of these parameters for the time horizons 2022 and 2027. This will indicate the evolution of the implementation of the circular economy model on the campus of the University of Lome.

## 2. Materials and Methods

### 2.1. Urban Solid Waste Generation at the University of Lome

The data were taken during a three-month stay in Togo. These data show the quantity and characteristics of waste currently generated on the campus.

To carry out the data collection, places for waste to be characterized were chosen in a way that guaranteed a representative sample. In this regard, in the case of faculties and schools, places with the highest numbers of students per campus area were chosen. This criterion was also considered in the case of residences and administrative centers, analyzing those with the largest numbers of workers and students.

**Figure 3:**
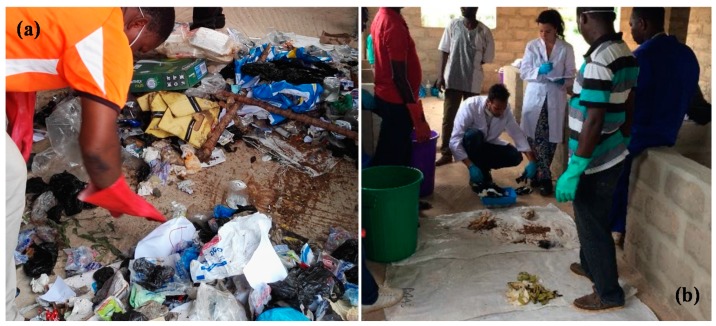
Laboratory work for urban solid waste characterization at the University of Lome. (**a**) Quartering and (**b**) weighing of some fractions after the separation.

As work methodology, a division of the work group into three teams was done. Each team was focused on a different area of the campus (north, center and south). Each of these teams was provided with plans with the location of the containers and work plan, as well as personal protective equipment (PPE). The procedure consisted of weighing and measuring the volume of each container, taking samples to be characterized by pouring them into bags prepared for this purpose, coding the bags to know which containers they came from, transferring the samples to the laboratory, reweighing to check the data previously taken, and proceeding with characterization (quartering them if necessary as observed in Figure 3a, separating them into different fractions that are studied in this work, weighing them with a precision scale as indicated in Figure 3b, and measuring the volume of each fraction with some containers of known volume).

Currently, 50,035 students are enrolled at the University of Lome, and 582 teachers and 1176 administrative officers work there. It also contains five restaurants and the second reference hospital in the country, with 300 beds. Due to the development level of the country, waste generated in the restaurants is considered urban solid waste and not as commercial waste. In the case of hospital’s waste, this study does not include health waste; since 2015 this hospital separates health waste and urban solid waste. In total, 1400 kg of urban solid waste were generated in the campus every day in 2018.

A circular economy model on the campus could be used to develop a similar one for the country, as the wastes generated are similar to those produced in the daily lives of people, independent of the place. This model is intended to serve as a reference for different institutions of the country, i.e., educational, social, and business infrastructures, regarding waste management. This is supported by a comparison of the percentages of different waste fractions obtained in this study, with the ranges indicated by Hoornweg and Bhada-Tata [20], with the only exception being the plastic fraction, as its percentage is higher in this work. This is probably due to the lack of drinking water on the campus, which implies that students have to buy water bags, which means an increase in the plastic fraction. Moreover, the evolution of the population has entailed a growth in the use of plastic containers over recent years. Currently, a cooperation project is analyzing the improvement of the drinking water supply system on the campus, which would allow for reducing the generation of plastic water bags almost entirely.

The waste collection that takes place on the campus is unitary: students, professors, and workers dispose of waste in single containers, without any type of triage. The implementation of an urban solid waste treatment plant would enable people to carry out manual triage every day, in order to take advantage of the fractions that can lead to a circular economy model.

The measurement of each fraction made it possible to know that the most common fractions generated on the campus are organic matter, paper and cardboard, textiles, plastics, and old tires:

➢ Organic matter generates bad odors and disease vectors, such as malaria. Composting and biogas can generate income for the university [28].
➢ Textile, paper, and cardboard are mostly from administrative and teaching centers. This fraction can be revalorized energetically [27].
➢ Plastic represents one of the highest fractions of global waste by mass nowadays. This trend goes along with the increasing environmental concern for post-consumer plastic waste [23]. Its sale and reuse can increase the income for the university.
➢ Old tires: the number of vehicles is increasing, resulting in an increase in tire waste at the end of their useful life [29]. The mixture of old crushed tires and sand can be used for road construction projects [30]. This can be particularly useful in order to carry out the next project of the university: to pave the streets of the campus.

**Figure 4:**
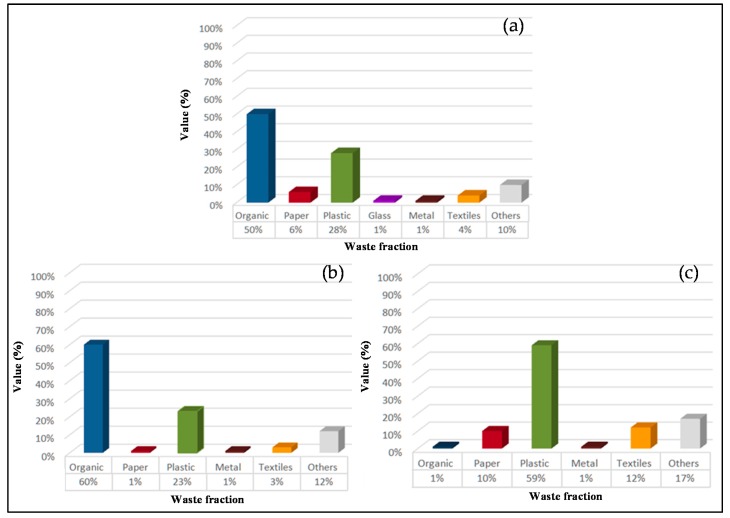
Real results obtained from quantification and characterization of urban solid waste generated at the University of Lome. (**a**) General composition of urban solid waste, (**b**) composition of waste from restaurants, (**c**) composition of waste from amphitheatres and classrooms.

Given the situation of poverty in the country and the purchasing power of students, the possibility of implementing circular economy indicators for glass and metal fractions was discarded, since they are almost non-existent (Figure 4a).

#### 2.1.1. Organic

Organic matter is composed of biodegradable components such as food scraps, yard waste (leaves, grass, brush), and wood.

Kouamé et al. [14] show that poor waste management leads to environmental threats and proliferation of disease. Organic matter is one of the biggest problems on the campus because it generates bad odors and disease vectors, like malaria.

According to Hoornweg and Bhada-Tata [20], low-income countries have the highest proportion of organic waste. Specifically, 695.5 kg of organic matter per day are generated on the university campus.

Most of this organic matter is generated in the 5 restaurants on campus (542 kg). Figure 4b shows the composition of waste from the restaurants (60% organic matter).

As Tchakpa et al. [31] show, composting production is the best option to introduce the organic fraction into the circular economy model, as compost can be used for the care of green areas. Biogas production would allow the university to generate energy.

#### 2.1.2. Paper

The paper fraction is composed of paper scraps, cardboard, newspapers, magazines, bags, boxes, wrapping paper, etc.

Paper waste could be used as fuel or for other applications. The paper industry is working on new technological solutions that allow adding value to this waste, converting it into resources for the papermaking process itself or for other industries. One of the solutions for waste paper is composting [32].

On the campus of the University of Lome, 79.5 kg of paper are generated per day, which can be added to the fraction of organic matter for composting.

#### 2.1.3. Textile

According to the Intergovernmental Panel on Climate Change [33], textile waste contains vegetable fibers that are slowly biodegradable. Consequently, it is possible to introduce them into the circular economy paradigm through composting, thus reducing the greenhouse gas emissions that would be produced by burning this waste fraction (currently, as the circular economy model has not been implemented yet, the burning of waste is usual on the campus).

On the campus of the University of Lome, 58 kg of textile waste are generated per day, which can be added to the fraction of organic matter for composting.

#### 2.1.4. Plastic

The fraction of plastic is composed of bottles, packaging, containers, bags, lids, cups, etc., constituting the second largest fraction that is generated. Consequently, the university community is concerned about the environmental impact of plastic waste on the campus, as accumulation of plastic waste is considered to be harmful to health [34].

Every day, 390 kg of plastic waste are generated on the campus. According to Salguero [34], this value will double in 10 years (2027). An average of 100 reusable 1.5 L bottles, whose mass is 32 g, are generated per day.

Figure 4c shows that amphitheaters and classrooms are the places where the greatest amount of plastic waste is generated.

There are two possibilities for plastic waste from the campus to enter the circular economy paradigm:

➢ Reusing bottles in good condition for the sale of soap and nuts.
➢ Selling the rest of the plastic in the port of Lome, where it can be used as energy fuel.

#### 2.1.5. Old Tires

In addition to the visual impact and the space occupied by the accumulation of tires, the partial chemical degradation the elements undergo converts tire graveyards into unsafe places. The environmental impact is greater when they end up on a field or in an uncontrolled landfill. In this case, they are dumped on the campus of the University of Lome. The accumulation of tires in landfills could lead to hazards such as uncontrolled fires, ideal habitats for disease-transmitting insects, and even the accumulation of water and leachate due to cavities that form in the internal face of this waste. The ecological catastrophes triggered by the accidental burning of unused tires in Hagersville (Ontario, Canada) and Seattle (Washington, USA) are widely known [35].

According to data from the Baobab Foundation (a Spanish foundation located on the campus of the University of Lome), 1 out of 100 campus inhabitants has a vehicle, so it is estimated that there are currently 520 vehicles on campus. Tire replacement is very common. This could possibly be the reason why old tires abound at uncontrolled scattered dumps.

Examples of circular economy with regard to building materials are increasingly popular [36]. Waste tire rubber is a promising lightweight aggregate for building products that enhances their properties [37].

According to Núñez-Cacho et al. [38], the construction industry is one sector that needs closer attention due to its environmental impact. An investigation developed by civil engineers at Purdue University used a mixture of old shredded tires and sand for road construction projects. Pieces of tires are an ecological alternative to more expensive materials, and are also easier to compact, saving time and money in construction [30]. This would lead to savings in applying asphalt to the campus road, which is currently unpaved.

### 2.2. Indicators of Circular Economy

The concern of the university community for conservation on the campus and the search for sustainable development in waste management have led to the implementation of environmental indicators with the following purposes:

➢ Indicators proposed here must serve as basic tools in the provision of information about the state of the environment, so that they contribute to awareness among public authorities and the population in general.
➢ They must be used in both the preparation and evaluation of environmental policies.

In this work, circular economy indicators are defined and calculated for each of the majority fractions described in the previous section, in order to reintroduce urban solid waste from the campus into the circular economy paradigm. Thanks to these indicators, the University of Lome can have a clear framework of the environmental situation on the campus, standardize the collection of generated waste data, manage environmental policies more easily, and perform regular comparative analyses about the generation of waste.

As Hu et al. [39] show, if the circular degree of waste economy on the campus can be estimated and the changes of some key indicators over time can be identified, it will be helpful in judging whether the university community is able to improve the campus situation.

#### 2.2.1. Organic, Paper, and Textile

The University of Lome has two options to include the organic, paper, and textile fractions into the circular economic paradigm: composting and biogas generation.

In recent years, due to strict regulations on waste landfilling in developed countries, anaerobic digestion of the organic fraction of municipal solid waste is increasingly being considered as a sustainable alternative for waste stabilization and energy recovery [40]. It can reduce the volume of waste and, at the same time, produce biogas and compost.

##### Composting

Composting is a natural aerobic process of biological stabilization of biodegradable waste that allows a reduction of mass and volume, and produces compost [41], which can be used for the care of green areas.

The indicator of circular economy efficiency for compost (I_c,ce_) provides information about the revalorization of organic matter, paper, and textile fractions as compost for green areas.

This indicator is defined according to Equation (1):

(1) $$I_{c,\mathrm{ce}}=\frac{m_{\mathrm{BW}}}{m_{T}}\cdot 100$$

where m_BW_ is the total amount of biodegradable waste generated for potential composting every day and m_T_ is the total amount of waste generated per day on the campus. This biodegradable waste includes organic matter and paper fractions, along with a small amount of textile waste.

This indicator can range between 0% and 100%: 0 means that no biodegradable waste is generated, and therefore no compost can be generated, whereas 100 means that all the waste generated every day is biodegradable, and therefore it can become compost.

##### Biogas

Biogas is a versatile renewable energy source consisting of up to 65% methane. Therefore, it could be used to replace fossil fuels in power and heat production [40].

The quantity of biogas generated depends on the composition and the quantity of waste, the infrastructure and the equipment available (compaction, insulation, collection and transport of leachates, cover system and periodic cover), and the design of the capture system that affects its efficiency [18].

Considering the uncertainty of the equipment that will be available on the campus, it will be assumed that internal combustion engine will be used. These engines are the most used, for their high efficiency and low cost per kW, compared to other systems [19].

From this perspective, the indicator of circular economy efficiency for biogas (I_b,ce_) provides information about the energetic revalorization of biodegradable waste.

This indicator is defined according to Equation (2):

(2) $$I_{b,\mathrm{ce}}=\frac{Q_{b}}{m_{T}}$$

where Q_b_ is the amount of biogas obtained from the anaerobic digestion of biodegradable waste generated every day on the campus.

This indicator shows the volume of biogas that is generated per kilogram of waste generated.

According to Consejería de Medio Ambiente y Ordenación del Territorio [42], it is possible to calculate the methane emitted by one ton of waste per year, as far as its composition is known (Equation (3)):

(3) $$L_{0}\left(\frac{m^{3}{\mathrm{CH}}_{4}}{t\;\mathrm{waste}}\right)=\mathrm{DOC}\cdot {\mathrm{DOC}}_{f}\cdot \frac{16}{12}\cdot \frac{1000}{0.656}\cdot \mathrm{MCF}\cdot F$$

where L_0_ is the methane generation potential per ton of waste, DOC is the amount of degradable organic carbon, DOC_f_ is the fraction of degradable organic carbon that actually decomposes (by default 0.5), MCF is the correction factor by anaerobic decomposition (equal to 1 for controlled landfills), and F is the volumetric fraction of CH_4_ in the generated biogas (by default 0.5).

Degradable organic carbon (DOC) depends on the composition of the waste removed. Each waste component has a different DOC value. It is calculated with Equation (4):

(4) $$\mathrm{DOC}=\underset{i}{\sum }{\mathrm{DOC}}_{i}\cdot W_{i}$$

where DOC_i_ is the percentage of degradable organic carbon contained in each waste fraction, which is 40% for paper, 24% for textile, and 15% for organic waste [42], and W_i_ is the percentage of each fraction with respect to the total biodegradable waste.

According to the Intergovernmental Panel on Climate Change [33], biogas is composed of approximately 50% methane (CH_4_) and 50% carbon dioxide (CO_2_). Consequently, the amount of biogas that can be generated from organic matter, paper, and textile fractions is twice the value of L_0_.

The volumetric flow of biogas obtained by the anaerobic digestion of biodegradable waste (Q_b_) is calculated according to Equation (5):

(5) $$Q_{b}\left(\frac{m³\;\mathrm{biogas}}{\mathrm{day}}\right)=\frac{2}{1000}\cdot L_{0}\cdot m_{\mathrm{BW}}$$

In addition, another indicator of circular economy efficiency for energy use of biogas (I_E,b,ce_) is defined according to Equation (6):

(6) $$I_{E,b,\mathrm{ce}}=\frac{E_{b}}{E_{c}}\cdot 100$$

where E_b_ is the energy produced on the campus every day thanks to biogas and E_c_ is the estimation of total energy consumed at the university.

This indicator provides information about how much energy from non-renewable sources could be replaced by energy from biogas, with a subsequent reduction in emissions of organic pollutants and greenhouse gases [26]. It can range between 0% and 100%: 0 means that no energy consumed can be replaced by energy from biogas, and 100 means that all the energy consumed from non-renewable sources can be replaced by energy from waste treatment in the form of biogas.

Each cubic meter of biogas contains around 6.5 kWh of energy, and the efficiency of biogas transformation into electric energy, for internal combustion engine, is considered to be 35%. The energy obtained from biogas every day on the campus (E_b_) is calculated with Equation (7):

(7) $$E_{b}\left(\frac{\mathrm{kWh}}{\mathrm{day}}\right)=6.5\cdot Q_{b}\cdot 0.35$$

According to Quirosa-Martin and Delgado-Ramos [43], the University of Lome campus consumes 308,034.00 kWh per month in each transformer (E). There are three transformers, so E_c_ is calculated with Equation (8):

(8) $$E_{c}\left(\frac{\mathrm{kWh}}{\mathrm{day}}\right)=\frac{3\cdot E}{30\;\cdot \;24}$$

#### 2.2.2. Plastic

There are two indicators of circular economy efficiency for plastic. They provide information about the reuse and revalorization of plastic fraction.

The first indicator is defined according to Equation (9), and it provides information about the quantity of plastic that can be reused in the form of bottles (I_pb,ce_):

(9) $$I_{\mathrm{pb},\mathrm{ce}}=\frac{m_{\mathrm{rp}}}{m_{p}}\cdot 100$$

where m_rp_ is the mass of reusable plastic generated every day on the campus, which is equal to the average mass of a plastic bottle multiplied by the number of bottles generated, and m_p_ is the total mass of plastic generated every day.

This indicator can range between 0 and values below 100%, indicating the reuse rate of the plastic fraction on the campus every day: 0 means that no plastic can be reused and 100 means that all plastic can be reused in the form of bottles.

In addition, the second indicator of circular economy for plastic fraction is defined according to Equation (10):

(10) $$I_{\mathrm{ps},\mathrm{ce}}=\frac{m_{\mathrm{sp}}}{m_{p}}\cdot 100$$

where m_sp_ is the mass of plastic that is sold per day.

This indicator provides information about the quantity of plastic that can be sold in the port of the city every day. There, plastic is energetically revalorized as energy fuel. If the value of this indicator is 0, it means that no plastic is generated on the campus that can be sold, whereas 100 means that all the generated plastic can be sold in the port, and therefore it is energetically revalorized.

#### 2.2.3. Old Tires

Waste tires are now considered as a source of valuable materials and structures that can be used to produce new goods and products of useful significance [29].

According to Centro de Estudios y Experimentación de Obras Públicas [44], rubber powder is obtained by crushing old tires. Incorporating rubber powder into a bituminous mixture modifies its rheological properties and improves its performance as a material to build roads.

The indicator of circular economy efficiency for old tires (I_ot,ce_) provides information about the reduction in raw material consumption to manufacture asphalt due to the contribution of old tire waste.

This indicator is defined according to Equation (11):

(11) $$I_{\mathrm{ot},\mathrm{ce}}=\frac{m_{\mathrm{rot}}}{m_{\mathrm{rn}}}\cdot 100$$

where m_rot_ is the mass of rubber contained in all existing tires on the campus, and m_rn_ is the total mass of rubber necessary to pave all the roads on the campus.

It is estimated that there are currently 520 vehicles on the campus.

With a tire mass of 8.6 kg and 45% rubber quantity in a tire [45], Equation (12) calculates the amount of rubber associated with old tires (m_rot_):

(12) $$m_{\mathrm{rot}}=4\cdot \mathrm{number}\;\mathrm{of}\;\mathrm{vehicles}\cdot \mathrm{tire}\;\mathrm{mass}\cdot \mathrm{rubber}\;\mathrm{fraction}$$

Taking into account the need to pave 121,200 m² of campus roads with asphalt [46] and the minimum necessary thickness of bituminous mixture is 15 cm [47], the volume of asphalt required can be calculated. According to Sol-Sánchez [48], who showed that rubber dust can replace 2% of the asphalt aggregate, the quantity of rubber necessary to pave the entire campus road (m_rn_) can be calculated according to Equation (13):

(13) $$m_{\mathrm{rn}}=0.02\cdot \mathrm{surface}\cdot \mathrm{thickness}\cdot \mathrm{bituminous}\;\mathrm{mixture}\;\mathrm{density}$$

### 2.3. Time Horizons 2022 and 2027

Salguero [34] estimated the waste that could be generated on the campus in 2022 and 2027, taking into account the evolution of the population, which tends to increase, and society, which tends to generate more waste per capita due to increased consumption. In addition to increased waste, the different fractions will also be modified due to consumption growth.

Based on the growth in the number of students at the University of Lome from 2006 until now, through an arithmetic method, Salguero [34] calculated that the number will increase from 50,035 students currently to 64,302 in 2022 and 78,569 in 2027.

Hoornweg and Bhada-Tata [20] show that the quantity of waste per capita generated in Togo will theoretically increase from 0.52 kg/(inhabitant·day) in 2012 to 0.85 kg/(inhabitant·day) in 2025.

Knowing the actual quantity of waste currently generated on the university campus each day, the increase in population, and the theoretical increase in the amount of waste generated per capita, Salguero [34] determined that the quantity of waste generated will be 2427.2 kg/day in 2022 and 3034.7 kg/day in 2027.

The evolution in humans’ way of living and eating means that the waste generated also changes over the years. As the population progresses, the percentages of waste fractions are modified; for example, the amount of plastic increases, while organic matter decreases [20]. Hoornweg and Bhada-Tata [20] show that the percentage of organic matter could decrease from 64% to 62% between 2012 and 2025 in developing countries, paper could increase from 5% to 6%, and plastic from 8% to 9%.

Knowing the actual percentage of each fraction of waste generated on the campus, the theoretical evolution and the theoretical increase of total waste generated in 2022 and 2027, Salguero [34] evaluated the theoretical quantity of each fraction generated in both time horizons.

This estimation allows some indicators presented in the previous section to be calculated in the future.

## 3. Results

### 3.1. Organic, Paper, and Textile

According to the Intergovernmental Panel on Climate Change [33], organic, paper, cardboard, and textile waste contain most of the DOC in urban solid waste. These fractions can be introduced into the circular economy thanks to composting and biogas generation.

#### 3.1.1. Composting

Compost, used as fertilizer, favors the activity of microbial life, prevents erosion and leaching of nutrients, and, in general, enhances and favors the biological activity of the soil, which is the best guarantee for preventing pests and diseases [49].

The organic matter contribution in soils in the form of compost positively affects the capture of CO_2_ and other greenhouse gases, thus helping to mitigate the effects of global warming [50].

According to Danso et al. [51], there is great potential to close the biodegradable waste loop, supporting the circular economy on the campus, and converting it into organic fertilizers.

Table 1 shows the values necessary to calculate indicator I_c,ce_, which provides information about the revalorization of biodegradable waste fractions as compost for green areas on the campus.

So, using Equation (1), the value of the circular economy indicator I_c,ce_ is 59.5%. Hence, 59.5% of the waste generated on the campus can be introduced into the circular economy paradigm through composting.

Molina-Moreno et al. [26] reported that 4.75% of biofertilizer was produced from the anaerobic digestion of wastewater from the pig farming industry. Additionally, Molina-Sánchez et al. [27] determined that 39.7% of the sludge generated during the paper production process was recovered and reused as mineral load in base paper manufacturing. These values concerning circular economy efficiency are lower than those obtained in this work (59.5% of biodegradable waste destined for composting).

According to Jouhara et al. [41], compost achieves a 90% transformation in waste weight. Consequently, 749.7 kg of compost could be obtained.

#### 3.1.2. Biogas

Biogas is a gas produced through the anaerobic digestion of biodegradable waste. Microorganisms metabolize carbon from organic substrates within an oxygen-free environment, i.e., anaerobically [52].

Table 2 shows the values used to calculate both indicators of this section. It should be highlighted that Table 1 shows the values of biodegradable waste generation to calculate the circular economy indicator regarding biogas production.

Using Equations (2) to (5), the value of the circular economy indicator I_b,ce_ is 0.109 m³ biogas/kg waste, that is, per each kilogram of waste generated on the campus, 0.109 m³ of biogas is obtained. This enables the introduction of biodegradable waste into the circular economy paradigm through energy generation. From Equations (6) to (8), the value of I_E,b,ce_ is calculated as 27.0%. That is, 27.0% of the energy consumed on the campus can be replaced by clean energy obtained from biogas.

In this regard, Molina-Moreno et al. [26] determined that 5.33 m^3^ biogas was generated per 1 m^3^ pig manure treated, which supposed a reduction of 5.33% in natural gas consumption. This value is considerably lower than that obtained in this work (27.0%).

### 3.2. Plastic

There is a need for a commitment to proper management of the impact of plastic waste and effective environmental management on the campus. The indicators calculated below show the possibility of introducing plastic waste generated into the circular economy paradigm through reutilization and revalorization.

Table 3 shows the values of plastic waste generation to calculate circular economy indicators regarding the plastic fraction and the results of indicators.

Using Equation (9), the indicator I_pb,ce_ is obtained. It relates the number of reusable bottles and the total amount of plastic generated on the campus. The value obtained is 0.8%, that is, 0.8% of the fraction of plastic generated on the campus every day can be introduced into the circular economy paradigm through reuse. Many reusable plastic bottles are used to sell nuts at the market and at restaurants on the campus. As there are no available figures for the actual current reuse of plastic, these figures only refer to potential reuse.

The second indicator (I_ps,ce_) is calculated using Equation (10), and it shows the percentage of plastic waste fraction that can be sold in the port of Lome. The value of m_sp_ is equal to 386.8 kg/day, so the value of indicator I_ps,ce_ is 99.2%. That is, 99.2% of the plastic generated on the campus can be sold.

It can be concluded that 0.8% of the plastic generated on the campus can be reused and 99.8% can be revalorized.

According to Salguero [34], in the port, the plastic fraction is sold for €0.04/kg and used as energy fuel. Taking into account daily plastic generation, an income of €15.5/day is obtained.

### 3.3. Old Tires

The indicator of circular economy regarding old tires (I_ot,ce_) is calculated from Equation (11) to (13), and it provides information on the introduction of old tires into the circular economy paradigm. Table 4 shows the data used to calculate this indicator and the result.

Indicator I_ot,ce_ is equal to 1.5%. That is, 1.5% of the rubber necessary to pave the entire roadway of the campus can be replaced by the waste generated by tires currently on the campus.

Roads play a crucial role in transporting people and providing access to schools, services, residences, etc. However, their construction involves harmful environmental impacts due to the carbon emissions stemming from their bitumen content [53]. The formation of landfills would be avoided by using old tires in the asphalt.

According to the California Department of Transportation [54], the advantage provided by incorporating rubber is the environmental benefit. Hicks and Epps [55] listed a series of benefits for the use of rubber in bituminous mixtures: it avoids the accumulation of tires in uncontrolled landfills, reduces the noise emitted by vehicles, provides added value to the waste (an improvement in the properties of asphalt mixtures), increases resistance to plastic deformation, and reduces the cost due to increased lifetime and less maintenance.

In this regard, this research characterizes the waste according to its potential for reutilization in order to be constituted as future technological nutrients in other productive processes, and considers its management within the circular economy paradigm, which could be achieved if each of the majority fractions (organic, paper, textile, plastic, and old tires) were exploited. Thus, those responsible for the campus should design a scorecard of indicators that could facilitate the decision-making process to manage the current negative externalities with these waste products. In light of this, waste management should be considered as the use of technological nutrients within the circular economy paradigm that allow evaluating the progress of sustainability in terms of reduction, reutilization, and recycling of the waste generated, which will enable the conversion of the actual linear economy model to the circular economy model.

### 3.4. Temporal Evolution: Time Horizons 2022 and 2027

These two time horizons are justified, since it is not possible to predict waste evolution within a horizon longer than 10 years in a developing country, because the pace of evolution is unknown. The estimations of total waste for these horizons are 2427.2 kg/day in 2022 and 3034.7 kg/day in 2027.

**Figure 5:**
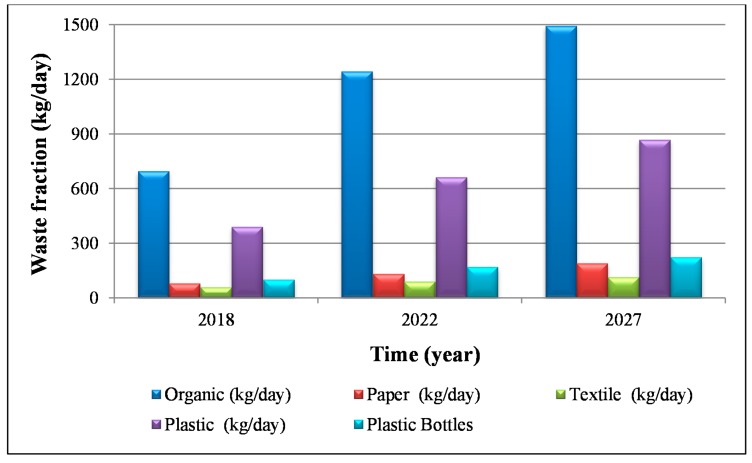
Quantity of each fraction currently generated on the campus, and time horizons 2022 and 2027.

**Figure 6:**
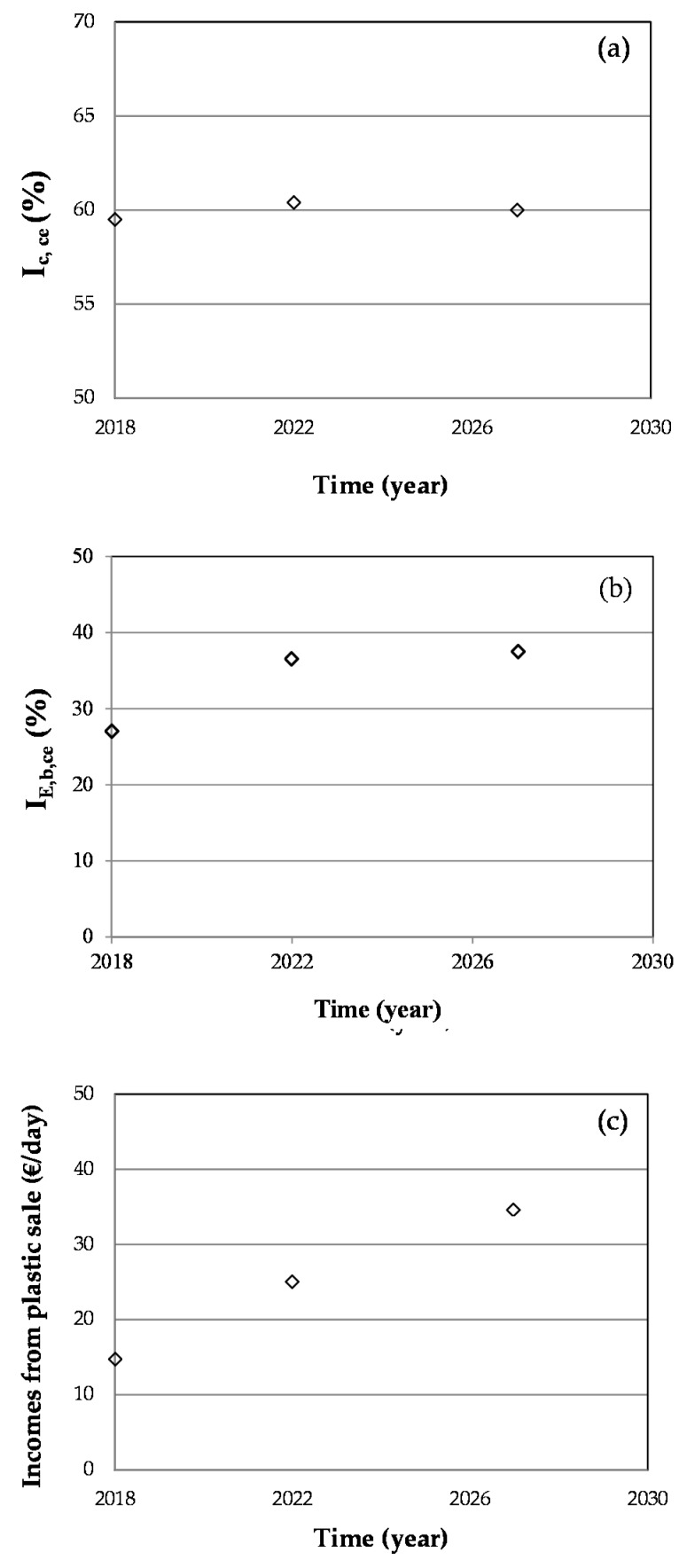
(**a**) Temporal evolution of percentage of biodegradable residue with respect to total residue generated on campus (I_c,ce_). (**b**) Temporal evolution of percentage of energy supplied by biogas with respect to energy consumed on campus (I_E,b,ce_). (**c**) Temporal evolution of income generated with the sale of plastic fraction in the city port.

Figure 5 shows the evolution of the different waste fractions. Taking into account this information, Figure 6 indicates the evolution of the composting and biogas indicators (I_c,ce_) and (I_E,b,ce_), and of the economic income obtained from the sale of the plastic fraction.

#### 3.4.1. Composting

Making use of Equation (1), the temporal evolution of the generation of biodegradable waste with respect to the total waste generated during each horizon year is obtained (Figure 6a). This evolution shows that in 2022, 60.4% of waste will be biodegradable, while in 2027, 60.0% will be biodegradable, and therefore could be used to generate compost.

The decrease in this percentage in 2027 is due to the fact that, although the biodegradable fractions are greater due to population growth, the plastic fraction has higher growth due to social development. This decrease does not mean that the amount of compost generated will be smaller, because it is directly proportional to the amount of biodegradable waste generated (organic, paper, and textile), and this increases.

#### 3.4.2. Biogas

By performing the same procedure as in subsection Biogas under Section 2.2.1, the temporal evolution of the indicator I_E,b,ce_ is obtained (Figure 6b). It is assumed that energy consumption will increase over the years according to the increase in population, which has been considered to elaborate Figure 6b.

This graph shows that 36.5% and 37.5% of the energy consumed on the campus could be obtained by the generation of clean energy (biogas) in 2022 and 2027, respectively. In this way, the University of Lome would introduce a large part of its waste into the circular economy paradigm and collaborate in conserving the environment.

#### 3.4.3. Plastic

As Figure 5 shows, the plastic fraction will increase in 2022 and 2027. Indicators regarding reusing and recycling the plastic fraction are calculated with respect to the total amount of plastic generated, so they do not change, because the amount of bottles generated in 2022 and 2027 is proportional to the amount of total plastic generated.

Figure 6c shows income variability from 2018 to 2027 due to the increase in the total amount of plastic generated.

The income from the sale of this fraction in 2027 will reach a value that is more than double the income from the current sale of plastic.

## 4. Conclusions

Production, management, and treatment of urban solid waste are among the most concerning aspects of sustainable development and environmental impact on the campus of the University of Lome. This causes two major negative externalities. On the one hand, it is a danger to the environment, and on the other hand, it is a threat to human health.

In this sense, this research, which was carried out through fieldwork on the university campus, achieves an important characterization that can serve as the basis for creating different circular economy indicators. These indicators can help provide those responsible for design with a new strategy to improve the management of the campus with regard to environmental and health benefits for the educational community.

In light of this, it should be highlighted that 59.5% of the waste generated on the campus in 2018 could be introduced into the sustainable waste management paradigm through composting. In addition, 27.0% of the energy consumed on the campus could be replaced by clean energy obtained from biogas in 2018. Regarding the plastic fraction, it could all be introduced into the circular economy paradigm by reusing plastic bottles and selling the rest in the port of the city. Thus, income obtained could range from €15.5/day in 2018 to €34.5/day in 2027. Concerning old tires, 1.5% of the rubber necessary to pave the entire roadway of the campus can be replaced by the waste generated by the tires currently existing on the campus.

Creating circular economy indicators can help this university create a specific program of zero waste on the campus, eliminating uncontrolled dumps. This also entails raising environmental awareness among the university community and the society of the city of Lome. In this regard, it should be noted that more business analysis and market studies are necessary for the correct implementation of a waste management plan based on circular economy model. Thus, future research lines could be developed to consider market requirements, logistics and other aspects of the business.

Consequently, this work aims to serve as a reference for the Togolese authorities, with the goal of creating legislative initiatives patterned on actions developed in Europe, China, and other African countries. As a result, those initiatives could be managed within the framework of the circular economy in order to reduce or remove the negative externalities that bad waste management entails, and to create a new and more sustainable production model that can serve as a reference in all productive sectors of Togo. In light of this, this specific strategy of waste management could comply with three objectives within the social responsibility programs of the University of Lome, i.e., improving health and environmental conditions on the campus, proposing research projects related to waste recycling and environmental sustainability, and promoting environmental awareness in the university community.

## Figures and Tables

**Table 1:** Circular economy indicator for composting.

Parameter	Value
Organic matter	695.5 kg/day
Paper	79.5 kg/day
Textiles	58 kg/day
Total biodegradable waste (m_BW_)	833 kg/day
Total urban solid waste (m_T_)	1400 kg/day
I_c,ce_	59.5%

**Table 2:** Circular economy indicators for biogas production. DOC, degradable organic carbon.

Parameter	Value
DOC	0.18
L_0_	91.46 m³ CH_4_/t waste
Biogas generated (Q_b_)	152.38 m³ biogas/day
Total urban solid waste (m_T_)	1400 kg/day
I_b,ce_	0.109 m³ biogas/kg waste
Energy obtained (E_b_)	346.66 kWh/day
Energy consumed (E_c_)	1283.48 kWh/day
I_E,b,ce_	27.0%

**Table 3:** Circular economy indicators for reutilization and revalorization of plastic fraction.

Parameter	Value
Mass of plastic waste (m_p_)	390 kg/day
Number of reusable plastic bottles (n_b_)	100 bottles/day
Medium bottle mass (m_am_)	32 g/bottle
Mass of reusable plastic (m_rp_)	3.2 kg/day
Mass of sold plastic (m_sp_)	386.8 kg/day
I_pb,ce_	0.8%
I_ps,ce_	99.2%

**Table 4:** Circular economy indicator for old tires.

Parameter	Value	Reference
Number of tires	2080 units	-
Tire mass	8.6 kg	Castro (2008)
Rubber in a tire	45 %	Castro (2008)
Mass of rubber contained in all existing tires on campus (m_rot_)	8049.6 kg	-
Surface to be paved	121,200 m²	Toura (2017)
Thickness of asphalt layer	15 cm	Ministerio de Fomento (2003)
Rubber in bituminous mixture	2 %	Sol-Sánchez (2011)
Bituminous mixture density	1500 kg/m³	-
Total mass of rubber necessary to pave campus roads (m_rn_)	545,400 kg	-
I_ot,ce_	1.5%	

## Nomenclature

Indicators of circular economy efficiency:

I_c,ce_	Compost (%)
I_b,ce_	Biogas (m^3^ biogas/kg waste)
I_E,b,ce_	Energy use of biogas (%)
I_pb,ce_	Reusable plastic (%)
I_ps,ce_	Sold plastic (%)
I_ot,ce_	Old tires (%)
